# Assembling the jigsaw puzzle: CBX2 isoform 2 and its targets in disorders/differences of sex development

**DOI:** 10.1002/mgg3.445

**Published:** 2018-07-11

**Authors:** Patrick Sproll, Wassim Eid, Camila R. Gomes, Berenice B. Mendonca, Nathalia L. Gomes, Elaine M.‐F. Costa, Anna Biason‐Lauber

**Affiliations:** ^1^ Division of Endocrinology University of Fribourg Fribourg Switzerland; ^2^ Department of Biochemistry Medical Research Institute University of Alexandria Alexandria Egypt; ^3^ Medical School University of Sao Paulo Sao Paulo Brazil

**Keywords:** CBX2, DamID, DSD, EMX2, gonad development

## Abstract

**Background:**

One of the defining moments of human life occurs early during embryonic development, when individuals sexually differentiate into either male or female. Perturbation of this process can lead to disorders/differences of sex development (DSD). Chromobox protein homolog 2 (*CBX2*) has two distinct isoforms, CBX2.1 and CBX2.2: the role of CBX2.1 in DSD has been previously established, yet to date the function of the smaller isoform CBX2.2 remains unknown.

**Methods:**

The genomic DNA of two 46,XY DSD patients was analysed using whole exome sequencing. Furthermore, protein/DNA interaction studies were performed using DNA adenine methyltransferase identification (DamID) to identify putative binding partners of CBX2. Finally, in vitro functional studies were used to elucidate the effect of wild‐type and variant CBX2.2 on selected downstream targets.

**Results:**

Here, we describe two patients with features of DSD i.e. atypical external genitalia, perineal hypospadias and no palpable gonads, each patient carrying a distinct CBX2.2 variant, p.Cys132Arg (c.394T>C) and p.Cys154fs (c.460delT). We show that both CBX2.2 variants fail to regulate the expression of genes essential for sexual development, leading to a severe 46,XY DSD defect, likely because of a defective expression of EMX2 in the developing gonad.

**Conclusion:**

Our study indicates a distinct function of the shorter form of CBX2 and by identifying several of its unique targets, can advance our understanding of DSD pathogenesis and ultimately DSD diagnosis and management.

## 1 INTRODUCTION

Disorder of sex development (DSD) combines a broad spectrum of different phenotypes, defined as congenital conditions in which the development of the gonad and/or anatomical sex is atypical. Current data indicate that approximately 1 in 4,500 birth exhibit genital anomalies, for which in only around 50% of cases a causal gene variant can be found (Hughes et al., 2006). One of the genes implicated in DSD is *CBX2/M33*, a member of the Polycomb group (PcG) proteins, that are conserved regulatory factors initially discovered in *Drosophila*, best known for their role in maintaining silent expression states of Hox genes during development by regulating chromatin structure and chromosome architecture at their target loci.

Knocking out M33 in mice affected the development of the genital ridge in both XX and XY embryos, suggesting that it functions upstream of Sry. The majority of XY mice showed sex reversal, whereas ovarian development was impaired in XX animals (Katoh‐Fukui et al., 1998). Cbx2 is able to activate Sf1 expression during spleen and adrenal development, suggesting that Cbx2 in mice has a role as transactivator, distinct from its chromatin modifier function (Katoh‐Fukui et al., 2006). Additionally, it has been shown that forced expression of Sry or Sox9 in Cbx2 KO mice could rescue to sex reversal in XY mice, although they presented with smaller gonads compared to WT mice (Katoh‐Fukui et al., 2012). Katoh‐Fukui et al. concluded that Cbx2 may regulate testis determination by regulating, directly or indirectly, Sry and additionally might influence gonadal size through the regulation of other genes. Our group made the discovery of a loss‐of‐function double heterozygote CBX2.1 variant in a 46, XY girl with ovarian‐like tissue at histology, normal uterus and external female genitalia, accidentally diagnosed because of a discrepancy between prenatal karyotype and phenotype at birth. Functional studies demonstrated that the CBX2.1 variant does not properly bind to and does not adequately regulate the expression of target genes essential for sex development such as *SF1/NR5A1*. Our data identified CBX2.1 as essential for normal human male gonadal development, suggested that it lies upstream of SRY in the human sex development cascade and identified a novel autosomal recessive cause of DSD. From a more mechanistic point of view, we demonstrated that CBX2.1 might have a role as transactivator, distinct from its known function as chromatin‐modifier (Biason‐Lauber, Konrad, Meyer, DeBeaufort, & Schoenle, 2009).

The function of the shorter isoform CBX2.2 is not yet well‐explored. To study this isoform, we investigated the c.394T>C (p.Cys132Arg) and the c.460delT (p.Cys154fs) CBX2.2 variants, from two individuals with 46, XY DSD, characterized by atypical external genitalia and dysgenetic testis.

## 2 MATERIALS AND METHODS

### 2.1 Subjects

The study was approved by our institutional ethics committee and written informed consent was given.

#### 2.1.1 Patient one

The first patient, a white 9 months‐old girl born at term and small for gestational age (2.300 g), was referred at the outpatient clinic of Hospital das Clínicas of University of Sao Paulo, with atypical external genitalia noticed at birth, characterized by microphallus (2.5 cm), perineal hypospadias and absence of palpable gonads. The karyotype was 46, XY. No Müllerian derivatives were found at pelvic ultrasonography and retrograde uretrocistography showed a blind vagina. At 2 years of age, a human chorionic gonadotropin (hCG) stimulation test was performed (two doses of 2,000 U) and no testosterone increase and steroid precursor accumulation was found. Since childhood, the patient showed a male behavior and after psychological evaluation, changed to male social sex at 5 years of age. He had a normal mental development. At 10 years of age he was submitted to exploratory laparotomy which disclosed bilateral atrophic testis that were removed. Anatomopathological data showed dysgenetic testes characterized by immature tubules with Sertoli cells only and a few atypical spermatogonias. No Leydig cells were identified in the interstitium. By 17 years of age, he started androgen replacement with testosterone esters. At this time his penile size was 12 × 3 cm, serum LH level was 16 U/L, FSH level was 54 U/L, and testosterone level was 230 ng/dl 14 days after exogenous testosterone (NV LH: 1,4–9,2 UI/L; FSH: 1,0–12 UI/L. Total testosterone: 271–965 ng/dL).

#### 2.1.2 Patient two

The second patient was a white 34 years of age male at the time of referral to the outpatient clinic of Hospital das Clínicas of University of Sao Paulo. He presented with atypical genitalia, characterized by microphallus (5.0 cm), bilateral cryptorchidism, perineal hypospadias and scrotum biphidus. 46, XY karyotype was confirmed by chromosome analysis and laboratory evaluation showed hypergonadotropic hypogonadism with LH levels of 10 U/L, FSH levels of 78 U/L, and testosterone levels of 75 ng/dL. A gonadectomy was performed, which revealed no gonadal tissue on histology evaluation and furthermore, a hypoplastic uterus was found. Genitoplasty, followed by testicular prosthesis implant was performed. In the meantime, testosterone replacement was started by using testosterone cypionate. The final penile length was 10 cm.

### 2.2 Whole exome sequencing

Genomic DNA samples from both patients were analysed by whole exome sequencing (WES) on a SureSelect Human All Exon V.6.0 capture kit (Agilent, Santa Clara, California, USA) and a HiSeq 4000 Sequencer (Illumina, San Diego, California, USA). The reads were screened with FastQ Screen (Babraham Bioinformatics, Cambridge, UK) for possible contamination and a quality control has been performed with FastQC (Babraham Bioinformatics). Read alignment has been performed with Bowtie 2 (Johns Hopkins University) against a human reference sequence (GRCh37.p13/hg19). GATK HaplotypeCaller (Broad Institute) was used for calling SNPs and indels, and the aligned data was visualized with the Integrative Genomics Viewer (Broad Institute). Genes with known variants causing similar phenotypes were screened first (e.g. *SRY*,* NR5A1/SF1, DMRT1*,* TCF21*,* GATA4*,* DHH*,* FGF9*,* FGFR2,* and *PGD2*). Then, a list of 85 genes known to be involved in 46,XY and 46;XX DSD in general, was used to further screen the WES data (Supporting information Table S1) (Baetens et al., 2017). Finally, additional potential candidate variants were selected based on occurrence in the general population (MAF < 0.5% in ExAC) and on the predicted influence on the protein level (SIFT, PolyPhen, Meta‐SNP, PhD‐SNP and SNAP).

### 2.3 Genome‐wide analysis of CBX2.2 binding sites

In order to study the functional consequences of the variants, we first had to identify potential targets of CBX2.2 in NT2/D1 (ATCC CRL‐1973) Sertoli‐like cells. Even though they are pluripotent cells, these cells express most of the important Sertoli‐cell markers (including SOX9, SF1 etc.) (Knower et al., 2007). To analyse protein/DNA interaction we used the DamID‐seq method that couples next‐generation sequencing to DamID (DNA adenine methyltransferase identification) assay (Eid, Opitz, & Biason‐Lauber, 2015). DamID is a methodology independent of antibodies, fixation, chromatin, shearing and other technically challenging features of Chromatin Immuno Precipitation (ChIP), and is a powerful tool for the mapping of genomic binding sites of chromatin binding proteins (Vogel et al., 2006). Briefly, human CBX2.2 cDNA (Origene, Rockville, Maryland, USA) was amplified and cloned into a pENTR11 plasmid (Invitrogen, Carlsbad, California, USA), then recombined into the destination vector pLgw‐V5‐EcoDam‐RFC1 to generate pLgw‐V5‐EcoDam‐CBX2, using the Gateway LR Clonase II enzyme mix according to the manufacturer's directions (Invitrogen). Lentiviral packaging plasmids, pRSV‐Rev (Addgene, plasmid 12253), pCMV‐dR8.2 (Addgene, plasmid 8455) and pMD2.G (Addgene plasmid 12259) were obtained from Addgene. DamID was performed using a lentiviral transduction protocol as previously described (Vogel, Peric‐Hupkes, & van Steensel, 2007), using pLgw‐EcoDam‐V5‐CBX2 (EcoDam‐CBX2) or pLgw‐V5‐EcoDam (Dam‐only). EcoDam‐CBX2 or Dam‐only were transfected into HEK239T cells (ATCC CRL‐11268) together with the packaging plasmids (pRSV‐Rev, pCMV‐dR8.2, pMD2.G) using Metafectene (Biontex Laboratories, Munich, Germany). 48 hr later, the supernatant containing the lentivirus was harvested and added to NT‐2D1 cells with 1 mg/ml polybrene (Santa Cruz Biotechnology, Dallas, Texas, USA), in two consecutive rounds of transduction. Two days after the first infection, genomic DNA was isolated using the DNeasy blood and tissue kit (Qiagen, Hilden, Germany). The isolated genomic DNA was subject to DpnI digestion, ligation of DamID adaptors, and DpnII digestion. Samples without addition of DpnI or ligation adaptors served as negative controls. DpnII‐digested fragments were used as templates to amplify methylated genomic fragments. DNA libraries were prepared using the TruSeq DNA LT Sample Prep Kit (Illumina) and the libraries were sequenced on a HiSeq2000 sequencer (Illumina). Cells expressing free Dam protein served as a control to correct for the unspecific DNA accessibility by Dam alone and for sequencing biases.

### 2.4 Immunofluorescence

Immunofluorescence was performed as previously reported (Eid et al., 2010). Briefly, cells grown on cover slips were pre‐extracted for 5 min on ice using 25 mM HEPES (Sigma‐Aldrich, St. Louis, Missouri, USA) pH 7.4, 50 mM NaCl (Acros Organics, Geel, Belgium), 1 mM EDTA (Acros Organics), 3 mM MgCL_2_ (Sigma‐Aldrich), 300 mM sucrose (Sigma‐Aldrich), and 0.5% Triton X‐100 (Sigma‐Aldrich) before fixation in 4% (w/v) formaldehyde (Sigma‐Aldrich) in PBS (Thermo Fisher Scientific, Waltham, Massachusetts, USA) for 15 min at room temperature (RT). Cover slips were incubated overnight at 4°C with primary antibodies anti‐V5 antibody (Santa Cruz Biotechnology) and Alexa‐conjugated secondary antibodies (Invitrogen) for 1 hr at RT. The cover slips were mounted with Vectrashield (Vector Laboratories, Burlingame, California, USA) containing DAPI. Images were acquired using a Nikon eclipse Ni‐E microscope system.

### 2.5 Functional analysis of the CBX2.2 mutants

We generated the p.Cys132Arg and p.Cys154fs variant CBX2.2s by site directed mutagenesis of wild‐type cDNA (Origene), according to the manufacture's manual (Quickchange II Site‐Directed Mutagenesis Kit; Agilent).

The influence of WT and CBX2.2 variants on the expression of selected candidate genes was studied by means of quantitative real‐time PCR, performed with ABI StepOnePlus Real‐Time PCR system, PCR products were quantified fluorometrically using the KAPA SYBR FAST master mix (KAPA Biosystems, Wilmington, Massachusetts, USA). The reference mRNA from cyclophilin was used for data normalization. Primers sequence and qRT‐PCR conditions for CBX2 isoforms and putative targets are available upon request. All samples were run in triplicates and finally the normalized relative expression values ($2^{-\Delta \Delta C_{t}}$) of multiple independent experiments were plotted against the relative expression values of the empty vector which was set as 1. Unpaired *t*‐test was performed using GraphPad Prism version 6.07 for Windows (GraphPad Software, La Jolla, California, USA).

### 2.6 Gene‐ontology enrichment analysis

ToppCluster was used for GO‐enrichment analysis of CBX2.2 Dam‐ID target genes (Kaimal, Bardes, Tabar, Jegga, & Aronow, 2010). GO‐enrichment permits to analyse functional features of gene sets, clustering them by their involvement in pathways related to Molecular Function, Biological Process and/or Cellular Component. GO‐terms with *p*‐values ≤0.05 and more than five target genes associated to the corresponding GO‐term were defined as significant. CBX2.2 Dam‐ID target genes were clustered depending on GO‐terms and visualized using spring‐embed layout with Cytoscape v3.3.0 (Shannon et al., 2003).

## 3 RESULTS

### 3.1 Identification of CBX2.2 mutations

No variants in the coding sequence of genes potentially causing a similar 46, XY DSD phenotype were found in the WES data of both patients including *SRY*,* TCF21*,* GATA4*,* DHH,* and *PGD2*. Patient one carries a *DMRT1* variant (c.133T>A, p.Ser45Thr), a synonymous FGF9 variant (c.1284A>G, p.Ser149Ser) and a synonymous FGFR2 variant (c.1343A>G, p.Val232Val), all common in the general population and predicted to be tolerable (Table 1). The second patient carries the same *DMRT1*,* FGF9,* and *FGFR2* variants as patient one and two *SF1/NR5A1* variants (c.437G>C, p.Gly146Ala, and c.499C>T, p.Pro125Pro), which are also common in the general population and predicted to be tolerable (Table 1).

**Table 1:** Variants found by whole exome sequencing in genes known to be related in 46, XX and 46, XY DSD for both patients

	Genes	Zygosity	AA. Sub.	SIFT	PhD‐SNP	SNAP	Meta‐SNP	PolyPhen	ExAC (MAF)	rs ID
Common mutations	LEPR	Hom	Q223R	0.28	0.363	0.665	0.15	0.047	0.5103	rs1137101
	LHX4	Het	N328S	1	0.063	0.45	0.068	0.004	0.485	rs7536561
	KISS1	Het	P81R	0.28	0.016	0.61	0.075	0.972	0.3413	rs4889
	AKR1C4	Hom	Q250R	1	0.324	0.39	0.437	0.001	0.2055	rs17306779
	WWOX	Hom	A179T	0.39	0.221	0.385	0.38	0.139	0.4507	rs11545029
	AMH	Hom	V515A	0.77	0.044	0.345	0.044	0	0.9799	rs10417628
	FBLN2	Het	T854A	0.3	0.102	0.465	0.266	0.012	0.7364	rs9843344
	SRD5A1	Het	A39G	—	0.091	0.255	0.044	0.001	0.6079	rs248793
	MAP3K1	Hom	D806N	0.19	0.396	0.555	0.112	0.092	0.5984	rs702689
	MAP3K1	Hom	V906I	0.76	0.137	0.51	0.059	0	0.7648	rs832582
	PROP1	Het	A142T	0.66	0.087	0.345	0.104	0.001	0.5113	rs4072924
	ZFPM2	Het	A403G	1	0.016	0.37	0.1	0	0.1146	rs11993776
	ZFPM2	Het	A1055V	0.16	0.093	0.535	0.17	0.046	0.01771	rs16873741
	DMRT1	Het	S45T	0.12	0.078	0.48	0.058	0.024	0.2678	rs3739583
	HSD17B3	Het	G289S	1	0.448	0.495	0.239	0	0.073306	rs2066479
	FGFR2	Hom	V232V	—	—	—	—	—	0.7797	rs1047100
	FGF9	Hom	S149S	—	—	—	—	—	0.8398	rs9509841
Patient one	PBX1	Hom	G21S	0.12	0.106	0.356	—	0.049	0.2977	rs2275558
	AMH	Hom	S49I	0	0.206	0.625	0.137	0.068	0.7876	rs10407022
	FSHR	Het	S654N	0.77	0.164	0.49	0.127	0	0.5727	rs6166
	FSHR	Het	A281T	0.5	0.113	0.47	0.187	0.005	0.5446	rs6165
	HHAT	Het	S182N	0.13	0.78	0.725	0.71	0.986	0.1439	rs2294851
	INSL3	Hom	T60A	1	0.051	0.115	0.05	0	0.6451	rs6523
	CYP21A2	Het	R103K	1	0.153	0.62	0.313	0.005	0.3341	rs75730897
	POR	Het	A503V	0.37	0.164	0.435	0.215	0	0.3639	rs1057868
	CYP11B1	Het	R43Q	1	0.219	0.48	0.27	0	0.08037	rs4534
	MAMLD1	Hom	P334S	0.02	0.206	0.565	0.178	0.22	0.08321	rs41313406
	MAMLD1	Hom	N662S	0.19	0.202	0.635	0.213	0.007	0.1089	rs2073043
Patient two	FBLN2	Hom	N839K	0.74	0.299	0.28	0.166	0.627	0.001426	rs61731214
	RXFP2	Het	I580V	0.27	0.122	0.53	0.225	0.02	0.1487	rs17076657
	NOBOX	Het	K664R	0.63	0.059	0.48	0.048	0.003	0.02763	rs77802098
	ZFPM2	Het	S657G	0.28	0.023	0.680	0.102	0.031	0.01258	rs28374544
	NR5A1	Het	G146A	0.5	0.187	0.43	0.246	0.001	0.1183	rs1110061
	NR5A1	Het	P125P	—	—	—	—	—	0.02645	rs1110062
	BMP15	Hom	S5R	0.01	0.736	0.23	0.475	0.235	0.01587	rs113099187

Variants are sorted into common between both patients, variants specific for patient one and variants specific for patient two. The effects of the variants on the protein level were predicted by SIFT, PhD‐SNP, SNAP, Meta‐SNP, and PolyPhen. Scores predicting a damaging effect of the variant are colored in red. In order to determine the occurrence in the general population, ExAC was used (rare: MAF < 0.005).

**Figure 1:**
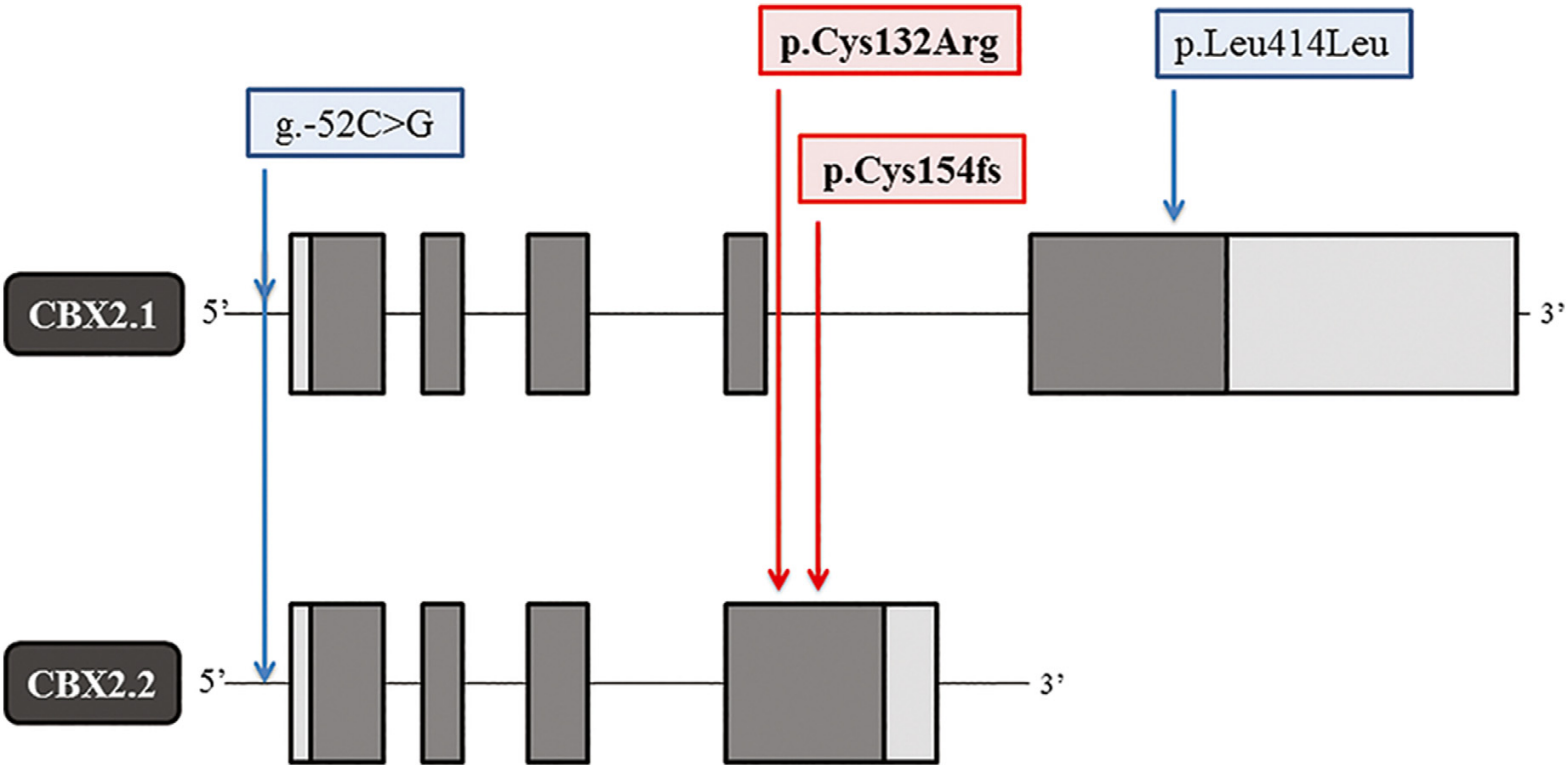
Schematic representation of the gene organization of both CBX2 isoforms. The CBX2.2 variants are depicted in red (Patient one: p.Cys132Arg; Patient two: p.Cys154fs). The two known SNPs found in CBX2.1, g.‐52C>G (rs71368052) and p.Leu414Leu (rs3751956), are depicted in blue

Screening the 85 genes implicated in 46, XY and 46XX DSD, revealed a c.394T>C variant in *CBX2.2*, leading to a p.Cys132Arg missense exchange in patient one and a c.460delT deletion, leading to a p.Cys154fs frameshift in patient two (Figure 1). The corresponding refSNP cluster ID numbers are rs782325368 and rs552892809 respectively. Both variants are rare, with a MAF on ExAC of 0.00001647 (p.Cys132Arg) and 0.001418 (p.Cys154fs) and are predicted to be disease causing. *CBX2.1* of patient one carries two known allelic variants (rs71368052 and rs3751956; Figure 1). ExAC predicts for *CBX2* a pLI (probability of LOF intolerance) of 0.95 (0 = gene tolerant to LOF; 1 = gene completely intolerant to LOF).

The WES data were further analysed to find other rare variants potentially causing the observed phenotype. Besides CBX2.2, there are 27 and 30 other gene variants in both patients, respectively, which were predicted to have a high to moderate impact (missense‐variants, frameshift‐variants, stop‐gained or splice acceptor/donor‐variants) and are rare (MAF < 0.5%) (Supporting information Tables S2 and S3). The unavailability of the parents DNAs prevented us from further filtering the variants. To analyze the putative relevance of these variants for the clinical phenotype, with the help of Pathway Studio 11 (Elsevier), we searched for connection between these genes with rare variants and female gonad development, male gonad development, gonad development, sex development, and sex determination. According to the literature, the only candidate gene implicated in these processes is CBX2, allowing us to conclude that the CBX2.2 variants are the most likely candidates for the observed phenotypes of the patients and prompted us to further analyze these variants.

### 3.2 Identification of CBX2.2 genomic targets

**Figure 2:**
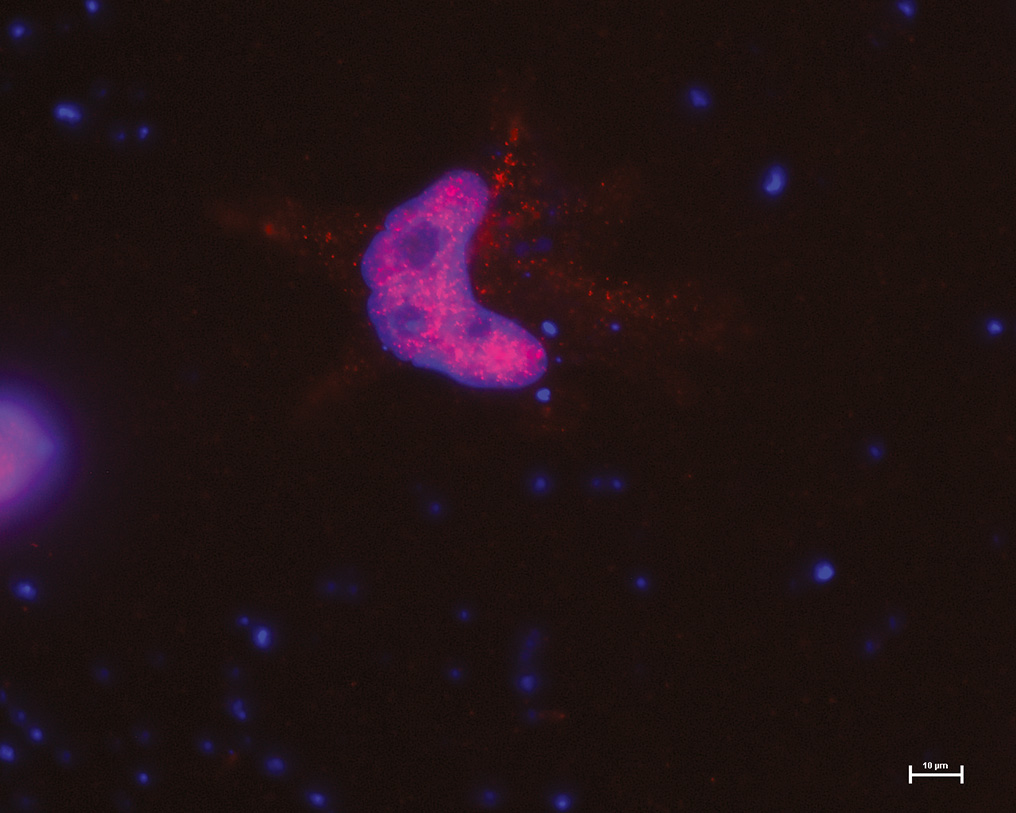
Dam‐CBX2.2 localization in NT2‐D1 cells. NT2‐D1 cells were transfected with the pLgw‐EcoDam‐V5‐CBX2 (Dam‐CBX2) and 48 hr later cells were detergent‐extracted and fixed. Dam‐CBX2 was visualized by indirect immunofluorescence using a V5 antibody, nuclei were visualized by DAPI. Bar 10 μm

To analyze the pathophysiological consequences of the CBX2.2 variants, we had to face the challenge of not knowing any targets for CBX2.2 and the consequent lack of functional assays. To gain insights into the function of CBX2.2 we applied the DamID‐seq method to identify its direct targets. We prepared two replicates for CBX2.1 and CBX2.2 fusion proteins or free Dam and sequenced the DNA libraries separately. Immunofluorescence analysis showed that the CBX2.2‐Dam construct correctly localized to the nucleus (Figure 2). All pairs of replicates had highly correlated read densities along the genome (Pearson correlation coefficients >0.90, *p*‐value <2e‐16) and thus were combined for the follow‐up analyses. Approximately 30% of CBX2.2 binding peaks were found in the promoter region, defined as 5Kb upstream of transcription start sites (Supporting information Figure S1). After normalizing the data to the controls, we identified 1901 enriched binding sequences of CBX2.2 within the promoter region of various genes, with a *p*‐value <1e‐5, of which 451 were shared with CBX2.1 and were excluded from further analysis. *SOX9* is one of the common targets and has also been excluded since it can be assumed that CBX2.1 might compensate for CBX2.2 deficiency. In silico bioinformatic analysis using Pathway Studio 11 (Elsevier) identified six potential CBX2.2 unique targets. Target selection was based on their reported putative involvement in sexual differentiation, their role in human disease and animal models or their specific expression in issues involved in sex development (gonads, sex organs, hypothalamus, and pituitary), as previously reported (Eid et al., 2015). Empty spiracles homeobox 2 (*EMX2)* (Simeone et al., 1992), Male germ‐cell associated kinase (*MAK)* which is exclusively expressed in testis and potentially important for spermatogenesis (Matsushime, Jinno, Takagi, & Shibuya, 1990), Homeobox A13 (*HOXA13*), a transcription factor which has been linked to syndromes affecting genitourinary development (Scott, Morgan, & Stadler, 2005), WD repeat‐containing protein 77 (*WDR77)* also known as androgen receptor‐associated protein, a 44‐kd protein interacting with Polycomb and a component of a methyltransferase complex involved in testis development and tumor formation (Liang et al., 2007), Twist Family BHLH Transcription Factor 1 (*TWIST1*), a HAT binding protein that also interacts with Polycomb proteins, was found to be related to androgen receptor expression (Shiota et al., 2010) and Basonuclein 2 (*BNC2*), which has been linked to hypospadias and distal urethral development (Bhoj et al., 2011). The analysis of the expression of these genes was used as an assay for WT versus mutant CBX2.2 function (see below).

### 3.3 Gene ontology analysis of CBX2.2 targets

**Figure 3:**
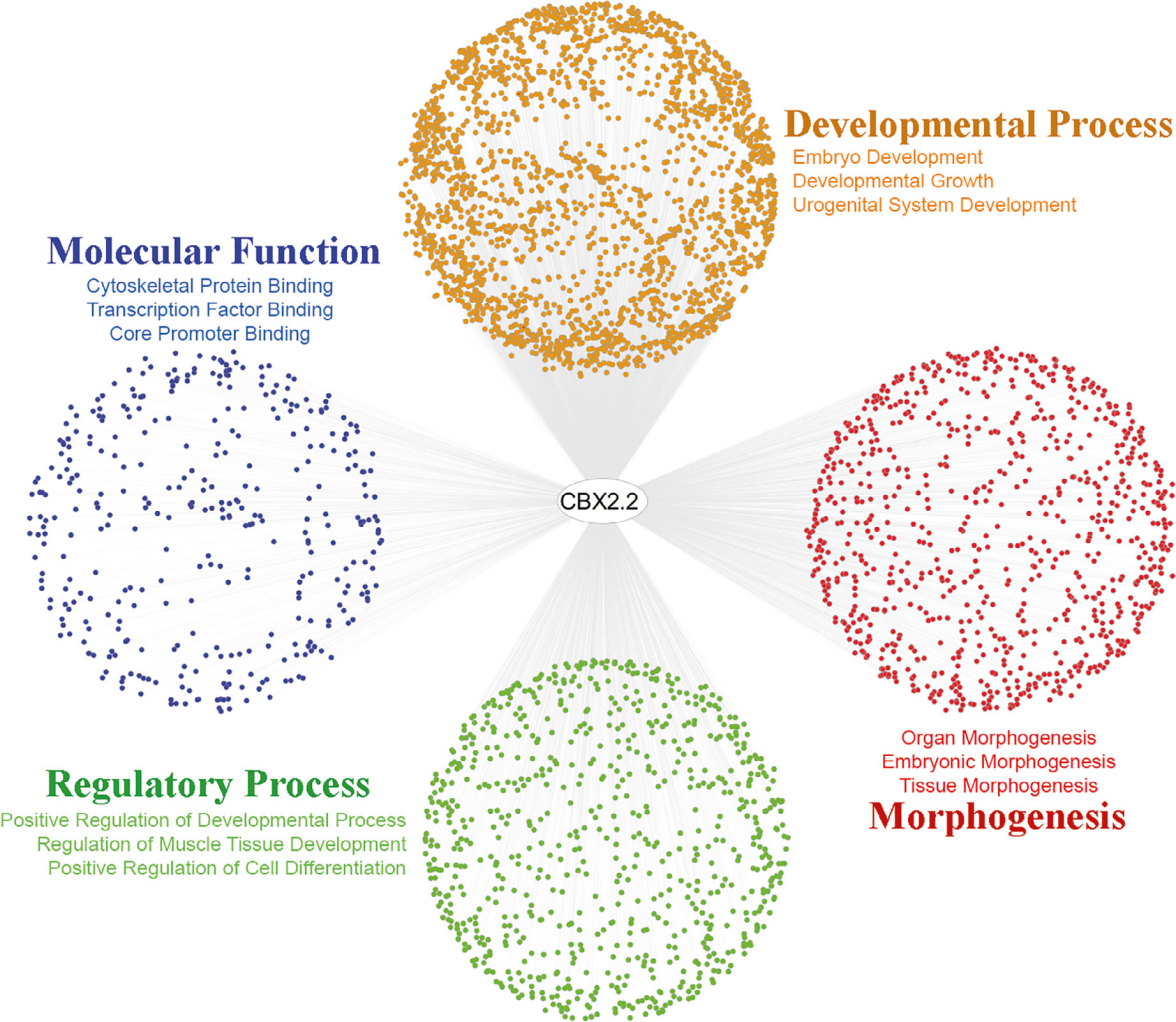
Representation of Gene Ontology (GO) enrichment analysis by Cytoscape. Every dot represents a gene related to the enriched GO‐terms, which have been clustered depending on their function. In orange are the GO‐terms represented which are involved in developmental processes, in red all GO‐terms related to morphogenesis, in green regulatory processes and in blue are the GO‐terms involved in molecular function

In order to retrieve a functional profile of the high‐throughput gene sets and therefore better understand the underlying biological processes, we performed an unbiased Gene Ontology enrichment analysis of the DamID targets. CBX2.2 targets showed an over‐representation of Gene Ontology terms related to development and morphogenesis (Figure 3), mainly in generic developmental terms like embryo development, heart development, and neuron development. Of particular interest for us, the analysis showed that the CBX2.2 targets are also overrepresented for the Gene Ontology term urogenital system development, additionally demonstrating the involvement of CBX2.2 in human sex development.

### 3.4 Functional characterization of CBX2.2 variants

**Figure 4:**
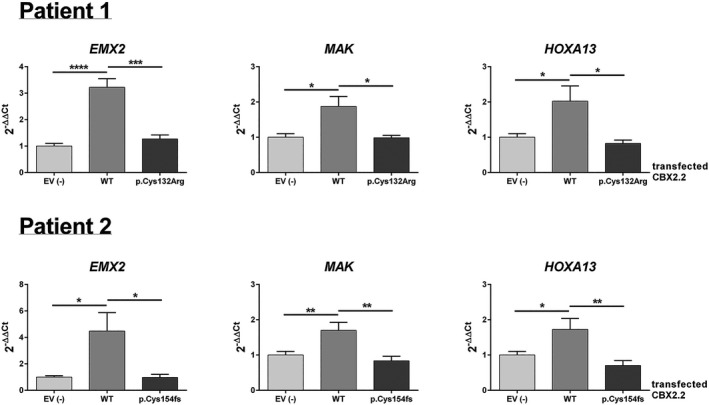
qRT‐PCR quantification of mRNA levels for the putative CBX2.2 targets EMX2, HOXA13, and MAK. NT2‐D1 cells were transfected with empty vector (EV), wild‐type (WT) CBX2.2 or CBX2.2 variants (p.Cys132Arg or p.Cys154fs) from patient number one or patient number two. Relative expression levels ($2^{-\Delta \Delta C_{t}}$) of target genes were determined by qRT‐PCR after normalization to cyclophilin as an endogenous control. Following WT CBX2 overexpression, the relative expression of EMX2, HOXA13 and MAK *,* was upregulated compared to cells transfected with empty vector (EV). Introducing either variant of CBX2.2 (p.Cys132Arg or p.Cys154fs) into the cells showed an effect comparable to negative control (i.e. no effect) on the expression of EMX2, HOXA13, and MAK, suggesting that failed regulation of these targets might contribute to the pathophysiology of the disease. The data in all graphs are the average of three independent experiments, error bars represent standard deviation from the mean (SEM) and values are expressed as relative to control = 1. (****) *p *<* *0.0001; (***) *p* < 0.001; (**) *p* < 0.01; (*) *p *<* *0.05

**Figure 5:**
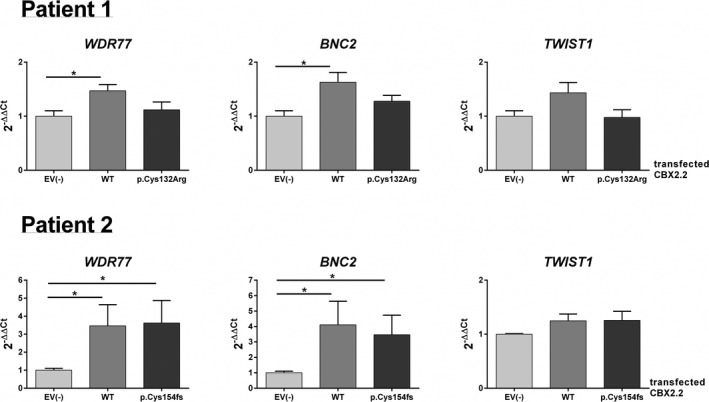
qRT‐PCR quantification of mRNA levels for the putative CBX2.2 targets WDR77, BNC2, and TWIST1. NT2‐D1 cells transfected with empty vector (EV), wild‐type (WT) CBX2.2 or CBX2.2 variants (p.Cys132Arg or p.Cys154fs) from patient number one or patient number two. Relative expression levels ($2^{-\Delta \Delta C_{t}}$) of target genes were determined by qRT‐PCR after normalization to cyclophilin as an endogenous control. Following WT CBX2 overexpression, the relative expression of WDR77 and BNC2 was up‐regulated compared to cells transfected with empty vector (EV). Both CBX2.2 variants had stimulatory effects similar to those of WT CBX2.2 on the expression level of WDR77, BNC2, and TWIST1, likely excluding them from the mechanism of disease. The data in all graphs are the average of three independent experiments, error bars represent standard deviation from the mean (SEM) and values are expressed as relative to control = 1. (*) *p *<* *0.05

To independently validate our findings, we performed functional studies using quantitative Real‐time PCR (RT‐qPCR) for the six selected targets. For the first variant (p.Cys132Arg), WT CBX2.2 increased the expression of EMX2, MAK, HOXA13, WDR77, and BNC in NT2‐D1 cells significantly (*p* < 0.05) by 3.2, 1.9, 2.0, 1.5, and 1.6, respectively, compared to the EV transfection (Figures 4 and 5). Using the CBX2.2 variant, the effect on EMX2, MAK, and HOXA13 significantly (*p* < 0.05) diminishes to expression values of 1.3, 1.0, and 0.8, with the expression of the target genes behaving essentially like the control, indicating that the effect is CBX2.2‐specific (Figure 4). Overexpression of the first CBX2.2 variant (p.Cys132Arg) increased the expression values for WDR77, BNC2, and TWIST1, compared to the EV, to 1.1, 1.3, and 1.0 respectively (Figure 5). However, they show no significant difference in expression under either WT or CBX2.2 variant expression. Similarly, by transfecting of the CBX2.2 variant found in the second patient (p.Cys154fs), the effect on the expression of EMX2, MAK, and HOXA13 also diminished significantly to expression values of 1.0, 0.8, and 0.7, again behaving essentially like the control (Figure 4). The expression values for WDR77, BNC2, and TWIST1 changed to 3.5, 3.5, and 1.3, respectively, when overexpressing the second CBX2.2 variant (p.Cys154fs) (Figure 5). Similar to the first variant, there is no difference in expression under either WT or the second CBX2.2 variant. The CBX2.2 variant mRNAs were expressed at a level similar to that of the wild‐type mRNA, suggesting that the variants do not influence mRNA stability. In order to ensure that the effect of CBX2.2 on EMX2 is isoform specific, we performed overexpression studies of CBX2.1 in NT2‐D1 cells. Overexpression of CBX2.1 showed no effect on EMX2 expression (Supporting information Figure S2).

## 4 DISCUSSION

Our patients represent the first two known variants in CBX2.2, the less known isoform of CBX2, associated to 46, XY DSD. In mice and men, loss‐of‐function of CBX2/M33, causes male‐to‐female and 46, XY DSD (MIM 613080) respectively. CBX2 isoform 1 was shown to be a transcription factor aside from its role as chromatin modifier and we could established that CBX2 isoform 1 regulates sex development by stimulating the male‐specific (e.g. *SF1* and *SOX9*) and inhibiting the female‐specific factors (such as *FOXL2*) (Biason‐Lauber et al., 2009; Eid et al., 2015). As for isoform 2, no function was known, mainly because of the lack of animal models, as there is no evidence of a CBX2.2 homologue in any other mammalian, including other primates, and the absence of human patients with CBX2.2 specific defects. The identification of the first such 46, XY DSD patients allowed us to investigate the function of CBX2.2.

Analysis of WES from the patients genomic DNA, showed no rare (MAF < 0.005) and potentially damaging variants related to 46, XY DSD, besides *CBX2* (Table 1). Furthermore, none of the 26 genes carrying rare variants (MAF < 0.005) in both patients and are predicted to have a high to moderate pathogenic impact, had any reported link to sex development or had previously been mentioned in DSD (Supporting information Tables S2, and S3) as analyzed using Pathway studio. This allows us to state that the CBX2.2 variants are the most likely cause for the observed phenotype of the patients.

For the identification of potential CBX2.2 binding targets and subsequent functional analysis of CBX2.2 variant function, we used the Sertoli‐like NT2/D1 cells, currently still the best available human cell model to study Sertoli‐cell development.

Using whole genome protein/DNA interaction and next generation sequencing approaches, we identified a large number of direct and unique potential targets of CBX2.2. Of all the CBX2.2 specific targets, the most relevant for sex development seems to be *EMX2*. *EMX2* is a paired‐like homeobox transcription factor, homologue of the Drosophila empty spiracle gene (Simeone et al., 1992). In the mouse, *Emx2* is essential for the development of several organs/tissues, such as the kidney and the urogenital system (Miyamoto, Yoshida, Kuratani, Matsuo, & Aizawa, 1997) and the central nervous system (CNS) (Galli et al., 2002; Gangemi et al., 2001; Heins et al., 2001; O'Leary, Chou, & Sahara, 2007). The absence of neurological impairment usually associated to *EMX2* variants (Granata et al., 1997) or *EMX2* haploinsufficiency (Piard et al., 2014) in our patients, might suggest that CBX2.2 regulates EMX2 expression specifically in the developing gonad and not the CNS. *Emx2* XY KO mice have gonadal agenesis (Miyamoto et al., 1997) due to proliferative defects of the coelomic epithelium, which gives rise to Sertoli cells. This implicated Emx2 in polarity of epithelial cells and their transition to mesenchymal cells, most likely through EGFR downregulation (Kusaka et al., 2010). Emx2 expression is stimulated by Wnt and Bmp signalling in nervous system development (Theil, Aydin, Koch, Grotewold, & Ruther, 2002) and it is repressed by HOXA10 during development of the endometrium of mice and humans (Daftary & Taylor, 2004; Troy, Daftary, Bagot, & Taylor, 2003) but very little is known about the control of *EMX2* in the developing gonad. In patients, deletions encompassing *EMX2* cause 46,XY DSD ranging from hypospadias to gonadal dysgenesis (Piard et al., 2014), confirming its role in human gonadal development (MIM 269160). In human development, EMX2 and CBX2 are simultaneously expressed in the gonadal anlage at 7 weeks of gestation, i.e. prior to the expression of SRY and testis determination, suggesting a role in the formation of the early gonad (Ostrer, Huang, Masch, & Shapiro, 2007). Subsequently, CBX2 is highly expressed in both sexes at 8 GW (but higher in female than male) then slightly decreases in male until basal, but still detectable expression level at 18 GW (E. Lecluze, F. Chalmel, INSERM‐Rennes, personal communication). In agreement with our data, Emx2 was also found downregulated in Cbx2‐deficient mouse gonads (Katoh‐Fukui et al., 2012). Based on our results, it is intriguing to hypothesize that CBX2.2 variants that impair expression of EMX2 might lead to severe defects of gonadal development in 46, XY individuals, similar to those individuals with *EMX2* haploinsufficiency (Piard et al., 2014) and might explain the severe testicular dysgenesis phenotype of the present CBX2.2 deficient patients, different from the ovarian‐like gonadal phenotype found in the 46,XY DSD CBX2.1 deficient patient (Biason‐Lauber et al., 2009).

Another target of CBX2.2 is HOXA13, a homeobox protein whose variants lead to and hand‐foot‐genital syndrome (also known as hand‐foot‐uterinesyndrome) or the related Guttmacher syndrome, dominantly inherited conditions characterized by Müllerian duct fusion defects of varying degree in females and hypospadias in males (Mortlock & Innis, 1997; Scott et al., 2005) (MIM 140000). *Hoxa13* mutant mice show loss of Fgf8 and Bmp7 expression and a decrease of the androgen receptor in male genital organs (Morgan, Nguyen, Scott, & Stadler, 2003) implicating *Hoxa13* in male sex differentiation and might partly explain the patients’ phenotype. This suggests that the mechanism of sex disorder in these CBX2.2‐deficient patients might affect the sex developmental processes primarily through impaired sex/gonadal development due to failure to regulate *EMX2*.

Our data suggest that both CBX2 isoforms are important for human sex development but they apparently have different functions, as shown by the difference in gonadal phenotype between loss‐of‐function of CBX2.1, which leads to residual gonadal development (Biason‐Lauber et al., 2009) and loss‐of‐function of CBX2.2, which leads to the here described 46, XY gonadal dysgenesis. DamID‐Seq analysis underlines the difference in function, since CBX2.1 and CBX2.2 only share about 25% of direct binding sequences.

There are other examples of the involvement of multiple isoforms in gonad development, the most prominent one being Wilms Tumor 1 (WT1). *WT1* codes for different isoforms, most important for gonad development are WT1−KTS and WT1+KTS, which differ only by the three amino acids Lysine (K), Threonine (T) and Serine (S). It has been shown that WT−KTS is required for gonad primordium survival, while the +KTS form seems mainly involved in the determination of the male pathway (Wilhelm & Englert, 2002).

The same appears to be the case for *CBX2*, as each isoform presents with distinct functions during gonad development. While CBX2.2 seems to be important for early bipotential gonad development, CBX2.1 seems to be mainly involved in testis determination. Nothing is known about the transcriptional control of *CBX2*, which could further help to elucidate the reason behind the different function of the isoforms and their spatiotemporal expression. However, alternative splicing and the existence of multiple isoforms with different functions during sex development and development in general, appear to be an important mechanism to control gene/protein cascades and differentiation pathways. Functional studies and analysis of spatiotemporal expression‐differences for isoforms could expand our current knowledge about sex development and further characterization of how *CBX2* and its targets fit into an ever expanding sex developmental network can impact our understanding of DSD pathogenesis and ultimately DSD diagnosis and management.

## CONFLICT OF INTEREST

No conflict of interest declared.

## References

[mgg3445-bib-0001] Baetens, D. , Stoop, H. , Peelman, F. , Todeschini, A. L. , Rosseel, T. , Coppieters, F. , … Cools, M. (2017). NR5A1 is a novel disease gene for 46, XX testicular and ovotesticular dosorders of sex development. Genetics in Medicine, 19, 367–376. 10.1038/gim.2016.118 27490115 PMC5392598

[mgg3445-bib-0002] Bhoj, E. J. , Ramos, P. , Baker, L. A. , Garg, V. , Cost, N. , Nordenskjöld, A. , … Zinn, A. R. (2011). Human balanced translocation and mouse gene inactivation implicate Basonuclin 2 in distal urethral development. Europen Journal of Human Genetics, 19, 540–546. 10.1038/ejhg.2010.245 PMC3083624 21368915

[mgg3445-bib-0003] Biason‐Lauber, A. , Konrad, D. , Meyer, M. , DeBeaufort, C. , & Schoenle, E. J. (2009). Ovaries and female phenotype in a girl with 46, XY karyotype and mutations in the CBX2 gene. American Journal of Human Genetics, 84, 658–663. 10.1016/j.ajhg.2009.03.016 19361780 PMC2680992

[mgg3445-bib-0004] Daftary, G. S. , & Taylor, H. S. (2004). EMX2 gene expression in the female reproductive tract and aberrant expression in the endometrium of patients with endometriosis. Journal of Clinical Endocrinology and Metabolism, 89, 2390–2396. 10.1210/jc.2003-031389 15126568

[mgg3445-bib-0005] Eid, W. , Opitz, L. , & Biason‐Lauber, A. (2015). Genome‐wide identification of CBX2 targets: Insights in the human sex development network. Molecular Endocrinology, 29, 247–257. 10.1210/me.2014-1339 25569159 PMC5414760

[mgg3445-bib-0006] Eid, W. , Steger, M. , El‐Shemerly, M. , Ferretti, L. P. , Peña‐Diaz, J. , König, C. , … Ferrari, S. (2010). DNA end resection by CtIP and exonuclease 1 prevents genomic instability. EMBO Reports, 11, 962–968. 10.1038/embor.2010.157 21052091 PMC2999859

[mgg3445-bib-0007] Galli, R. , Fiocco, R. , De Filippis, L. , Muzio, L. , Gritti, A. , Mercurio, S. , … Vescovi, A. L. (2002). Emx2 regulates the proliferation of stem cells of the adult mammalian central nervous system. Development, 129, 1633–1644. 11923200 10.1242/dev.129.7.1633

[mgg3445-bib-0008] Gangemi, R. M. , Daga, A. , Marubbi, D. , Rosatto, N. , Capra, M. C. , & Corte, G. (2001). Emx2 in adult neural precursor cells. Mechanisms of Development, 109, 323–329. 10.1016/S0925-4773(01)00546-9 11731244

[mgg3445-bib-0009] Granata, T. , Farina, L. , Faiella, A. , Cardini, R. , D'Incerti, L. , Boncinelli, E. , & Battaglia, G. (1997). Familial schizencephaly associated with EMX2 mutation. Neurology, 48, 1403–1406. 10.1212/WNL.48.5.1403 9153481

[mgg3445-bib-0010] Heins, N. , Cremisi, F. , Malatesta, P. , Gangemi, R. M. , Corte, G. , Price, J. , … Götz, M. (2001). Emx2 promotes symmetric cell divisions and a multipotential fate in precursors from the cerebral cortex. Molecular and Cellular Neuroscience, 18, 485–502. 10.1006/mcne.2001.1046 11922140

[mgg3445-bib-0011] Hughes, I. A. , Houk, C. , Ahmed, S. F. , Lee, P. A. , LWPES Consensus Group; ESPE Consensus Group (2006). Consensus statement on management of intersex disorders. Archives of Disease in Childhood, 91, 554–563. 10.1542/peds.2006-0738 16624884 PMC2082839

[mgg3445-bib-0012] Kaimal, V. , Bardes, E. E. , Tabar, S. C. , Jegga, A. G. , & Aronow, B. J. (2010). ToppCluster: A multiple gene list feature analyzer for comparative enrichment clustering and network‐based dissection of biological systems. Nucleic Acids Research, 38, W96–W102. 10.1093/nar/gkq418 20484371 PMC2896202

[mgg3445-bib-0013] Katoh‐Fukui, Y. , Miyabayashi, K. , Komatsu, T. , Owaki, A. , Baba, T. , Shima, Y. , … Morohashi, K. (2012). Cbx2, a polycomb group gene, is required for Sry gene expression in mice. Endocrinology, 153, 913–924. 10.1210/en.2011-1055 22186409

[mgg3445-bib-0014] Katoh‐Fukui, Y. , Owaki, A. , Toyama, Y. , Kusaka, M. , Shinohara, Y. , Maekawa, M. , … Morohashi, K. (2006). Mouse Polycomb M33 is required for splenic vascular and adrenal gland formation through regulating Ad4BP/SF1 expression. Blood, 106, 1612–1620. 10.1182/blood-2004-08-3367 15899914

[mgg3445-bib-0015] Katoh‐Fukui, Y. , Tsuchiya, R. , Shiroishi, T. , Nakahara, Y. , Hashimoto, N. , Noguchi, K. , & Higashinakagawa, T. (1998). Male‐to‐female sex reversal in M33 mutant mice. Nature, 393, 688–692. 10.1038/31482 9641679

[mgg3445-bib-0016] Knower, K. C. , Sim, H. , McClive, P. J. , Bowles, J. , Koopman, P. , Sinclair, A. H. , & Harley, V. R. (2007). Characterisation of urogenital ridge gene expression in the human embryonal carcinoma cell line NT2/D1. Sexual Development, 1, 114–126. 10.1159/000100033 18391522

[mgg3445-bib-0017] Kusaka, M. , Katoh‐Fukui, Y. , Ogawa, H. , Miyabayashi, K. , Baba, T. , Shima, Y. , … Morohashi, K. (2010). Abnormal epithelial cell polarity and ectopic epidermal growth factor receptor (EGFR) expression induced in Emx2 KO embryonic gonads. Endocrinology, 151, 5893–5904. 10.1210/en.2010-0915 20962046

[mgg3445-bib-0018] Liang, J. J. , Wang, Z. , Chiriboga, L. , Greco, M. A. , Shapiro, E. , Huang, H. , … Lee, P. (2007). The expression and function of androgen receptor coactivator p44 and protein arginine methyltransferase 5 in the developing testis and testicular tumors. Journal of Urology, 177, 1918–1922. 10.1016/j.juro.2007.01.017 17437848

[mgg3445-bib-0019] Matsushime, H. , Jinno, A. , Takagi, N. , & Shibuya, M. (1990). A novel mammalian protein kinase gene (mak) is highly expressed in testicular germ cells at and after meiosis. Molecular and Cellular Biology, 10, 2261–2268. 10.1128/MCB.10.5.2261 2183027 PMC360573

[mgg3445-bib-0020] Miyamoto, N. , Yoshida, M. , Kuratani, S. , Matsuo, I. , & Aizawa, S. (1997). Defects of urogenital development in mice lacking Emx2. Development, 124, 1653–1664. 9165114 10.1242/dev.124.9.1653

[mgg3445-bib-0021] Morgan, E. A. , Nguyen, S. B. , Scott, V. , & Stadler, H. S. (2003). Loss of Bmp7 and Fgf8 signaling in Hoxa13‐mutant mice causes hypospadia. Development, 130, 3095–3109. 10.1242/dev.00530 12783783

[mgg3445-bib-0022] Mortlock, D. P. , & Innis, J. W. (1997). Mutation of HOXA13 in hand‐foot‐genital syndrome. Nature Genetics, 15, 179–180. 10.1038/mg0297-179 9020844

[mgg3445-bib-0023] O'Leary, D. D. , Chou, S. J. , & Sahara, S. (2007). Area patterning of the mammalian cortex. Neuron, 56, 252–269. 10.1016/j.neuron.2007.10.010 17964244

[mgg3445-bib-0024] Ostrer, H. , Huang, H. Y. , Masch, R. J. , & Shapiro, E. (2007). A cellular study of human testis development. Sexual Development, 1, 286–292. 10.1159/000108930 18391539

[mgg3445-bib-0025] Piard, J. , Mignot, B. , Arbez‐Gindre, F. , Aubert, D. , Morel, Y. , Roze, V. , … Van Maldergem, L. (2014). Severe sex differentiation disorder in a boy with a 3.8 Mb 10q25.3‐q26.12 microdeletion encompassing EMX2. American Journal of Medical Genetics, 164A, 2618–2622. 10.1002/ajmg.a.36662 24975717

[mgg3445-bib-0026] Scott, V. , Morgan, E. A. , & Stadler, H. S. (2005). Genitourinary functions of Hoxa13 and Hoxd13. Journal of Biochemistry, 137, 671–676. 10.1093/jb/mvi086 16002988

[mgg3445-bib-0027] Shannon, P. , Markiel, A. , Ozier, O. , Baliga, N. S. , Wang, J. T. , Ramage, D. , … Ideker, T. (2003). Cytoscape: A software environment for integrated models of biomolecular interactions networks. Genome Research, 13, 2498–2504. https://doi.org/0.1101/gr.1239303 14597658 10.1101/gr.1239303 PMC403769

[mgg3445-bib-0028] Shiota, M. , Yokomizo, A. , Tada, Y. , Inokuchi, J. , Kashiwagi, E. , Masubuchi, D. , … Naito, S. (2010). Castration resistance of prostate cancer cells caused by castration‐induced oxidative stress through Twist1 and androgen receptor overexpression. Oncogene, 29, 237–250. 10.1038/onc.2009.322 19802001

[mgg3445-bib-0029] Simeone, A. , Gulisano, M. , Acampora, D. , Stornaiuolo, A. , Rambaldi, M. , & Boncinelli, E. (1992). Two vertebrate homeobox genes related to the Drosophila empty spiracles gene are expressed in the embryonic cerebral cortex. EMBO Journal, 11, 2541–2550. 1352754 10.1002/j.1460-2075.1992.tb05319.x PMC556729

[mgg3445-bib-0030] Theil, T. , Aydin, S. , Koch, S. , Grotewold, L. , & Ruther, U. (2002). Wnt and Bmp signalling cooperatively regulate graded Emx2 expression in the dorsal telencephalon. Development, 129, 3045–3054. 12070081 10.1242/dev.129.13.3045

[mgg3445-bib-0031] Troy, P. J. , Daftary, G. S. , Bagot, C. N. , & Taylor, H. S. (2003). Transcriptional repression of peri‐implantation EMX2 expression in mammalian reproduction by HOXA10. Molecular and Cellular Biology, 23, 1–13. 10.1128/MCB.23.1.1-13.2003 12482956 PMC140663

[mgg3445-bib-0032] Vogel, M. J. , Guelen, L. , de Wit, E. , Peric‐Hupkes, D. , Loden, M. , Talhout, W. , … van Steensel, B. (2006). Human heterochromatin proteins form large domains containing KRAB‐ZNF genes. Genome Research, 16, 1493–1504. 10.1101/gr.5391806 17038565 PMC1665633

[mgg3445-bib-0033] Vogel, M. J. , Peric‐Hupkes, D. , & van Steensel, B. (2007). Detection of in vivo protein‐DNA interactions using DamID in mammalian cells. Nature Protocols, 2, 1467–1478. 10.1038/nprot.2007.148 17545983 PMC7032955

[mgg3445-bib-0034] Wilhelm, D. , & Englert, C. (2002). The Wilms tumor suppressor WT1 regulates early gonad development by activation of Sf1. Genes & Development, 16, 1839–1851. 10.1101/gad.220102 12130543 PMC186395

