# Supplementary material for: Assembling the jigsaw puzzle: CBX2 isoform 2 and its targets in disorders/differences of sex development
Source: Mol Genet Genomic Med. 2018 Jul 11;6(5):785–95. doi: 10.1002/mgg3.445 (PMC6160712; doi:10.1002/mgg3.445)

**Supporting Information**

**Figure S1.** Enriched CBX2.2 binding regions representing different genomic features. Genomic locations up to 5Kb upstream of the transcription start site (TSS), are defined as the promoter region.


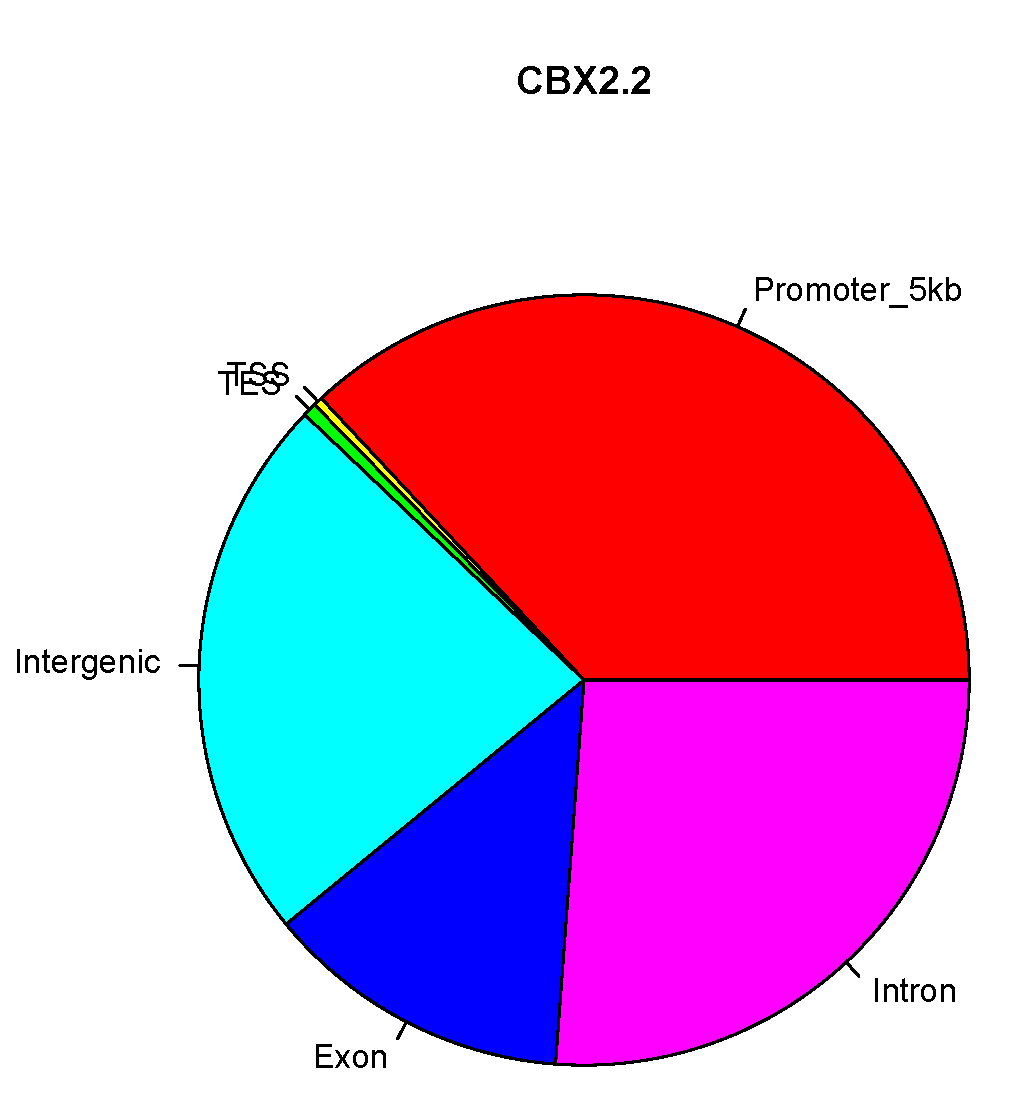


**Figure S2.** Overexpression of CBX2.1 has no effect on the expression of EMX2 in NT2D1 Sertoli-like cells. The data in all graphs are the average of three independent experiments, error bars represend standard deviation from the mean (SEM) and values are expressed as relative to control = 1.

**

**

**Table S1.** Table of 85 genes, which are known to be involved in 46,XY and/or 46,XX disorders of sex development. This table was used for filtering of the patient’s WES data.

| AKR1C1 | CTNNB1 | DMRT1 | FOXL2 | HSD3B2 | LHX9 | PROK2 | SRD5A2 |
| --- | --- | --- | --- | --- | --- | --- | --- |
| AKR1C2 | CYB5 | EAP1 | FSHR | INSL3 | LHβ | PROKR2 | SRY |
| AKR1C4 | CYP11A1 | EMX1 | FSHβ | KAL1 | MAMLD1 | PROP1 | STAR |
| AMH | CYP11B1 | EMX2 | GATA4 | KISS1 | MAP3K1 | PSMC3IP | TSPYL |
| AMHR2 | CYP17A1 | FAM58A | GnRHR | LEP | NELF | RSPO1 | WDR11 |
| AR | CYP19A1 | FBLN2 | GPR54 | LEPR | NOBOX | SEMA3A | WNT4 |
| ARX | CYP1B2 | FGF8 | HESX1 | LGR8 | NR5A1 | SOX2 | WT1 |
| ATRX | CYP21A2 | FGF9 | HHAT | LHCGR | NR5A2 | SOX3 | WWOX |
| BMP15 | DAX1 | FGFR1 | HOXA13 | LHX1 | NR0B1 | SOX8 |  |
| CBX2 | DHCR7 | FKBP4 | HSD17B1 | LHX3 | PBX1 | SOX9 |  |
| CHD7 | DHH | FOG2 | HSD17B3 | LHX4 | POR | SRD5A1 |  |

**Table S2.** Rare variants found by WES in patient one. Variants, which are also present in patient two are underlined. The effect of the variants on the protein level was predicted by SIFT, PhD-SNP, SNAP, Meta-SNP and PolyPhen. Scores predicting a negative effect are coloured in red. ExAC was used to determine the occurrence in the general population (rare: MAF<0.005).


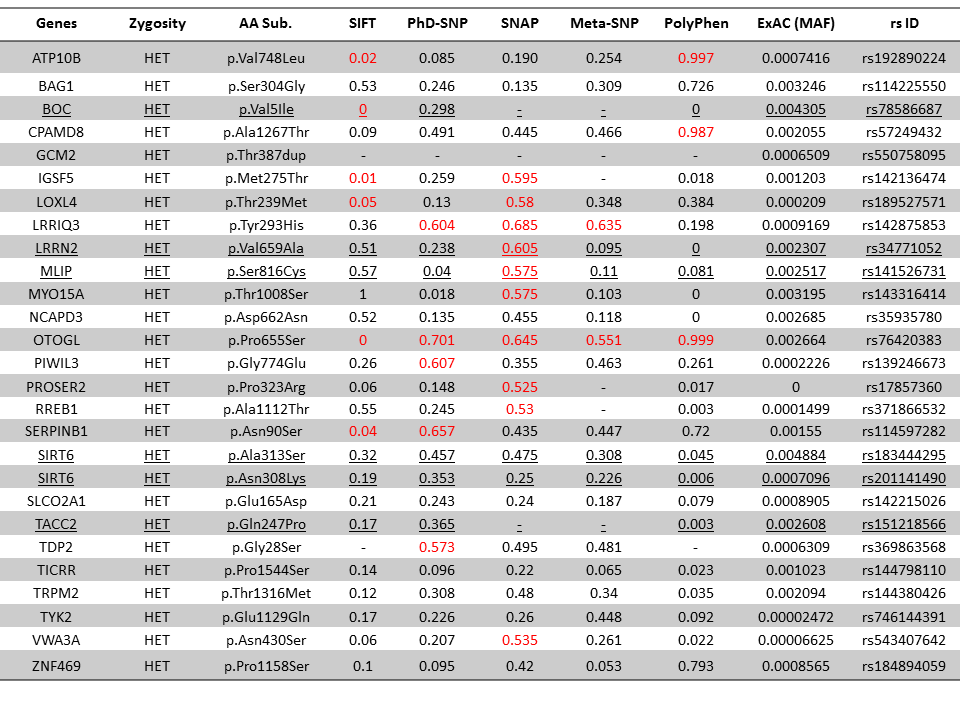


**Table S3.** Rare variants found by WES in patient two. Variants, which are also present in patient one are underlined. The effect of the variants on the protein level was predicted by SIFT, PhD-SNP, SNAP, Meta-SNP and PolyPhen. Scores predicting a negative effect are coloured in red. ExAC was used to determine the occurrence in the general population (rare: MAF<0.005).


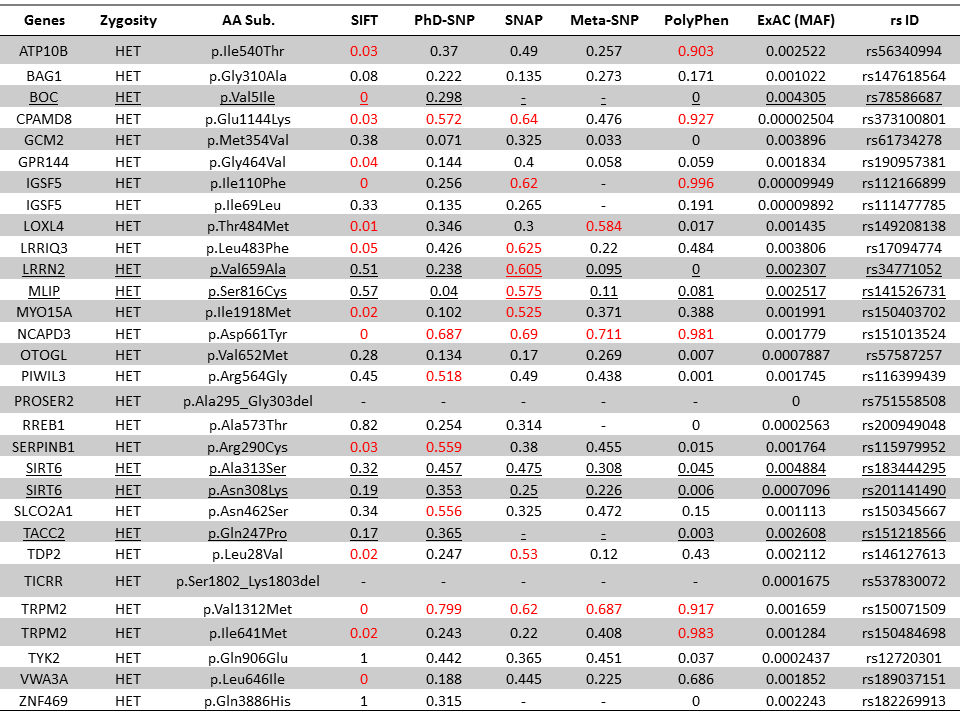

Supplement: Supplementary file 1 [file MGG3-6-785-s001.docx]
